# A study protocol for the randomized controlled trial SAFIR FAMILY TALK: a selective primary preventive intervention vs. service as usual for children of parents with mental illness

**DOI:** 10.1186/s13063-023-07256-6

**Published:** 2023-04-22

**Authors:** Signe S. Nielsen, Lisbeth J. Mikkelsen, Nikolaj Quaade, Tracy R. G. Gladstone, William R. Beardslee, Katrina Bonnemose, Nicole K. Rosenberg, Carsten Hjorthøj, Anne A. E. Thorup, Merete Nordentoft, Anne Ranning

**Affiliations:** 1 Mental Health Center Copenhagen, Research Unit (CORE), Copenhagen, Capitol Region of Denmark Denmark; 2 Wellesley Centers for Women, Wellesley, MA USA; 3 Boston’s Children’s Hospital, Boston, USA; 4 Mental Health Centre Copenhagen, Copenhagen, Denmark; 5 Department of Public Health, University of Copenhagen, Copenhagen, Denmark; 6 Department of Clinical Medicine, Faculty of Health and Medical Sciences, University of Copenhagen, Copenhagen, Denmark; 7 Child and Adolescent Mental Health Center, Copenhagen, Capital Region of Denmark Denmark; 8 Department of Psychology, University of Copenhagen, Copenhagen, Denmark

**Keywords:** Children of parents with mental illness, Prevention, Parental mental illness, Family Talk Preventive Intervention, Transgenerational transmission, Randomized controlled trial

## Abstract

**Background:**

Children of parents with mental illness have an increased risk of developing mental illness themselves throughout their lifespan. This is due to genetic factors but also environmental disadvantages during childhood associated with parental mental illness. Selective primary preventive interventions for the children are recommended to mitigate risk factors and strengthen protective factors, but large-scale, longitudinal studies are needed. This study aims to investigate the effect of the Family Talk Preventive Intervention in a cohort of children and their parents with mental illness.

**Methods:**

The study is a randomized controlled trial with 286 planned families with at least one parent with any mental illness and at least one child aged 7 to 17 years. It will be carried out in the mental healthcare system in the Capital Region of Denmark. Families will be referred from hospitals and municipalities. The children and parents will be assessed at baseline and then randomized and allocated to either the Family Talk Preventive Intervention or service as usual. The intervention group will be assigned to Family Talk Preventive Intervention, a manualized programme consisting of ~ seven sessions for the family, including psychoeducation about parental mental illness and resilience in children, stimulating dialogue between family members and creating a common family narrative. The study period for both groups will be 12 months. Follow-up assessments will be conducted after 4 months and 12 months. The primary outcomes are the children’s level of functioning, parental sense of competence and family functioning.

**Discussion:**

Given the prevalence of transgenerational transmission of mental illness, a systematic approach to prevention is needed in the mental healthcare setting. This study provides valuable knowledge on the Family Talk Preventive Intervention with a large sample size, inclusion of any parental mental illness and examination of the primary outcomes.

**Trial registration:**

ClinicalTrials.gov NCT05615324. Registered on 26 October 2022. Retrospectively registered.

## Administrative information

This protocol was written in accordance with the Standard Protocol Items: Recommendations for clinical Interventional Studies (SPIRIT) guidelines [1]. Note: the numbers in curly brackets in this protocol refer to SPIRIT checklist item numbers. The order of the items has been modified to group similar items (see http://www.equator-network.org/reporting-guidelines/spirit-2013-statement-defining-standard-protocol-items-for-clinical-trials/).

**Table:** 

Title {1}	A study protocol for the randomized controlled trial SAFIR FAMILY TALK: a selective primary preventive intervention vs. service as usual for children of parents with mental illness.
Trial registration {2a and 2b}.	ClinicalTrials.gov. Unique Protocol identifier: NCT05615324 Date of registration: 26–10-2022
Protocol version {3}	4.0, 13–1-2022
Funding {4}	This trial is funded by Trygfonden; ID: 127,849, and by Tværspuljen; ID: P-2021–1-13.
Author details {5a}	Signe S. Nielsen ^1^, Lisbeth J. Mikkelsen^1^, Nikolaj Quaade^1^, Tracy R. G. Gladstone^2^, William R. Beardslee^3^, Katrina Bonnemose^1^, Nicole K. Rosenberg^4^, Carsten Hjorthøj^1,5^, Anne A. E. Thorup^6,7^, Merete Nordentoft^1,6^, Anne Ranning^1,8^. 1: Mental Health Center Copenhagen, Research Unit (CORE), Capitol Region (DK). 2: Wellesley Centers for Women, Massachusetts, (USA). 3: Boston’s Children’s Hospital, (USA). 4: Mental Health Centre Copenhagen, Copenhagen (DK). 5: University of Copenhagen, Department of Public Health (DK). 6: Department of Clinical Medicine, Faculty of Health and Medical Sciences, University of Copenhagen (DK). 7: Child and Adolescent Mental Health Center, Capital Region of Denmark (DK). 8: University of Copenhagen, Department of Psychology (DK).
Name and contact information for the trial sponsor {5b}	Trygfonden: Hummeltoftevej 49, 2830 Virum. (DK) Phone number + 45 45 26 08 26. Sektion for Tværsektoriel Forskning: Nordre Fasanvej 57, 2000 Frederiksberg. (DK) Julie Grew Phone + 45 2628 5755.
Role of sponsor {5c}	The funders were not involved in the design of the trial, in writing the manuscript, or in the decision to submit the manuscript for publication. They will not be involved in the collection, management, analysis, or interpretation of the data. The primary investigator reports back with a trial status to Trygfonden on a yearly basis, and Tværspuljen every 6^th^ months from ultimo 2021-medio 2022.

## Introduction

### Background and rationale {6a}

An estimated two in five children grow up with a parent affected by mental illness [2, 3]. Children born to parents with severe mental illnesses like schizophrenia, bipolar disorder and major depressive disorder have an increased expectancy of developing a psychiatric disorder compared to the background population. By young adulthood, more than half have developed unspecific psychiatric disorders, and a third will have a severe mental illness [4]. Also during child and adolescent years, familial high-risk offspring show early signs of developmental disorders, anxiety, stress or adjustment disorders [5], and the risk of developing a psychiatric disorder is increased by a factor of 2–4 compared to children of parents without a mental illness [6].

### Family Talk Preventive Intervention

The idea of preventive intervention is not new, in fact, Family Talk was invented by William Beardslee and colleagues in the 1980s. In families affected by parental depression, the intervention has exhibited sustained improved effect after 4.5 years on parental child-related behaviours and attitudes, child-reported understanding of parental disorder and family functioning [7] and a reduction in internalizing symptoms in the children of parents with mood disorders [8]. Only one study has investigated Family Talk for families affected by parental mental illness of other diagnostic categories than mood disorders, including psychosis and bipolar and personality disorder, and reported high satisfaction with the intervention by both children and parents [9], thus supporting the assumption that Family Talk is safe and feasible in transdiagnostic psychiatric populations.

The intergenerational transmission of mental illness from parents to children may take place through a complex interplay of genetics, neurobiological risk factors [10] and a range of psychosocial factors. The latter may either be directly associated with the parents’ behaviour, cognitions and emotions or may emerge through a myriad of familial and contextual stressors associated with parental mental illness. Indeed, studies have shown higher incidence of factors such as family disruption, single-parent-headed households and parental unemployment [11], stigma and isolation [12], all influencing parental resources. Intrinsic factors in the child may also predispose them to be more or less affected by parental mental illness, including temperament, gender and cognitive and social skills [13]. They are less likely to graduate from primary school or achieve high grades [14], have a higher cumulative morbidity and mortality rate [15] and are to some extent overlooked by both the mental health services and social services of the municipality [16].

Preventive and supportive interventions are widely recommended, and a recent review in Lancet Psychiatry presented a mental health prevention strategy identifying children of parents with mental illness as a population, who, owing to their increased risk for mental illness alone, acquire selective primary preventive intervention to shift expected trajectories towards mental illness [17]. In recent years, Mental Health Services around the world have had an increased focus on development and evaluation of selective prevention initiatives. In Chile [18] and Greece [19], for example, randomized clinical trials are piloted for families with parental depression, following the original intent of the Family Talk Preventive Intervention. In Germany [20] and the Republic of Ireland [21], researchers are expanding the method to include parents with a range of psychiatric diagnoses.

### Objectives {7}

The main objective of this trial is to compare the Family Talk Preventive Intervention to service as usual for families with a parent(s) with any mental illness. Our primary outcomes are:

1. The child’s level of functioning
2. The parent’s sense of competence
3. Family functioning

We hypothesize that the Family Talk Preventive Intervention is superior to service as usual in the improvement of both the child’s and the parents’ well-being at the end of the intervention and after 12 months.

### Trial design {8}

The design of the trial is a two-armed, parallel, randomized trial testing for the superiority of the Family Talk group versus service as usual. The groups are allocated in a ratio of 1:1.

## Methods: participants, interventions and outcomes

### Study settings {9}

Participants are recruited from different psychiatric centres in the Capital Region of Denmark, together with their family members, from both inpatient and outpatient sites. In addition, families may be referred to the trial from Child Protection Services if inclusion criteria are met.

### Eligibility criteria {10}

#### Eligibility criteria for trial participants

Families are eligible if at least one parent meets the following criteria:

1. Parent(s) must have at least one ICD-10 [22] psychiatric diagnosis by a psychiatrist
2. At least one point of contact with the secondary mental health system within the previous 2 years before the assessment day
3. Have at least one child between the ages of 7 and 17 on the day of the assessment

Exclusion criteria exclude participants who do not speak Danish or English.

#### Eligibility criteria for trial interventionists

The intervention will be performed by mental health care professionals specifically trained in the Family Talk Preventive Intervention (nurses, psychologists, social workers, etc.). Training includes 100 h of instruction delivered over the course of 4 months, during which each clinician will conduct an intervention of their own with a family and receive supervision from an international expert in the Family Talk method. A written assignment on the intervention concludes the training period and will be evaluated by the expert for the clinician to be certified in the Family Talk method. The plan was that all training with the specialist would be face-to-face sessions, but due to COVID-19 some sessions were performed virtually. The re-scheduling of the training programme prolonged the training by approximately 1 month.

#### Who will take informed consent? {26a}

The assessors will obtain informed consent from all participants and children above the age of 15. The custodian(s) must consent to the children participating in the trial (if custody is shared, both parents need to give their informed consent).

Referred participants will undergo a process of informed consent which includes receiving written information regarding the type of intervention, potential risks/side effects and the study design. The researchers ensure that the patient fully understands the information given and accepts being randomly assigned to either the Family Talk intervention or service as usual. Participation in the study is voluntary, and the families can withdraw their consent at any time.

#### Additional consent provisions for collection and use of participant data and biological specimens {26b}

On the consent form, the patients can decide to accept being contacted in the future regarding the outcome of the study. Patients who decline this option still participate in the study. Patients can also decide whether the researchers can have access to their personal patient records. Furthermore, patients can give consent to recording of video and audio files, and if these can be used for educational purposes.

No biological specimen will be collected.

### Interventions

#### Background and rationale: choice of comparators {6b}

Service as usual according to the clinical guidelines of the Mental Health Services in the Capital Region of Denmark has been chosen as the comparison group. See below item 11a.

#### Intervention description {11a}

##### Service as usual (comparison group)

Service as usual is what would normally be offered to eligible families when the SAFIR trial is not running: According to the clinical guidelines of the Mental Health Services in the Capital Region of Denmark, all patients who are parents are to be offered a *next of kin conversation*, preferably at the beginning of their course of treatment. This is a conversation with the patient, their child(ren) and a mental health professional focusing on the parent’s mental illness and the child’s well-being. A preparatory session with the patient (and sometimes co-parent or other adult family member) precedes a session including both parent(s) and children. This session is informative in nature; the professional explains mental illness to the children, facilitates conversation between parents and children and may refer the family to other services such as support groups for children. If deemed necessary, a notification is sent to Child Protection Services who will then evaluate the need for further, statutory intervention for the child and family. The Mental Health Services train key workers to conduct *next of kin conversations* using a 10-day course that includes educational background knowledge about the needs of children of parents with mental illness as well as training in how to lead conversations with the families. There is a large variation in the level of awareness for patients’ children between different clinical units in the Mental Health Center, Capital Region, and the prevalence of *next of kin conversations* being offered. The service as usual arm of the study contains no intervention from this study group. Service as usual was chosen as a comparator to investigate the potential benefits of introducing a more comprehensive intervention like Family Talk into the secondary mental health sector as opposed to only one or two sessions of psychoeducation. Some patients may also be involved in services under Child Protection Services. Information on the families’ use of all relevant services focusing on parenting roles in relation to mental illness and their children is documented for both arms.

##### The intervention

**Fig. 1:**
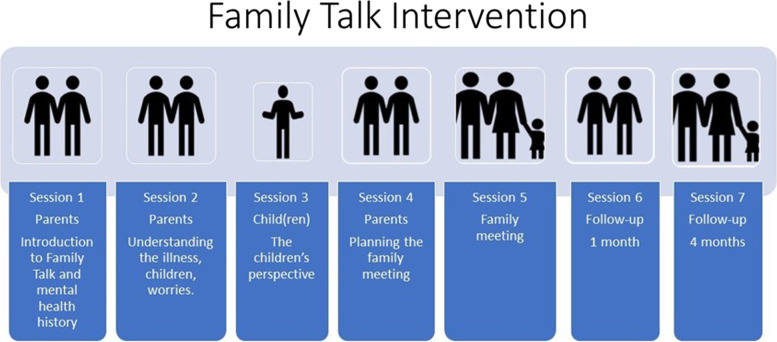
Family Talk Intervention. A schematic overview

Families in the *experimental intervention condition* will be offered approximately 7 sessions of the SAFIR Family Talk Preventive intervention. See Fig. 1. This is a clinician-facilitated, psychoeducational preventive intervention that includes on average 7 sessions designed to improve family communication and understanding of parental mental illness, improve interpersonal relationships and promote child resilience and utilization of social support [23]. An important tool throughout the intervention is the logbook which the clinician uses for taking notes with each family. The logbook prescribes the planned topics to be covered in each session and the contents of the sessions are noted in the logbook by the clinicians. Two modifications have been made to the original method. First, SAFIR Family Talk includes parents with any mental illness not only depression, and second, in the rare cases where the parent is incapacitated by mental illness and thus unable to participate in the intervention, the other parent and children are invited to participate.

Due to COVID-19, the clinicians were forced by hospital guidelines to wear facemasks and/or visors in some of the sessions during 2020 and 2021.

##### Description of core modules

*Module 1 and 2:* cover the parent’s history of mental illness, both parents’ view on how the child has been affected by the illness, and both parents’ view on the child’s daily life in the family, school and extracurricular domains. Any worries about the child(ren) are discussed. The unique and positively defining quality of the family is identified and verbalized, and the parents voice their desired goal of participating in the intervention. In *module 3* (one meeting per child): the clinician sees each child in the family individually and covers the child’s well-being and daily life in the family, school and extracurricular domains, any recurring conflicts in the family and any concerns about the parent’s illness. *Module 4*: Is a planning session with the parents, during which the parents receive feedback from the child meetings, and where a family meeting (Module 5) agenda is developed. *Session 5*: During this session, guided by the clinician, the parents put the mental illness into words and address any problem within the family. Furthermore, the clinician also helps the children to put forward any possible concern or issue. Two follow-up sessions conclude the intervention. *Session 6*: is a follow-up session after 1 month where the parents, together with the clinician, can talk about how they experienced the family session and the possible impact on the family dynamic. *Session 7*: takes place after a few months with the family discussing the intervention, the future for the family and whether further help is needed. The Family Talk Preventive Intervention lasts 6 weeks to 2 months, and each session has a duration of 60 min on average (also see Fig. 1).

Due to COVID-19, the intervention period was prolonged for some families receiving the intervention in 2020 and primo 2021.

#### Criteria for discontinuing or modifying allocated interventions {11b}

Family Talk is a flexible intervention regarding the number of sessions offered per family. If the clinician deems it necessary, an extra follow-up session or planning session with the parents is possible. Also, the clinician can call for a meeting with the family and social services, teachers or health personnel if further help or support is needed. The service as usual arm of the study contains no intervention from the study group.

#### Strategies to improve adherence to interventions {11c}

During the intervention, every session completed is documented by the clinician according to the contents of the logbook using REDCap electronic data capture tools [24]. Sessions are either recorded on audio or video. For each clinician, two randomly selected sessions will be rated, on a yearly basis, by an independent expert regarding fidelity, i.e. compliance with the specific components of the sessions as prescribed in the logbook and manual. The expert will also evaluate the competency of the clinician based on the intervention provided in the sessions, including generic psychotherapeutic factors. The expert will provide feedback on fidelity to each clinician based on the evaluations. In addition, all clinicians will have comprehensive experience with psychotherapeutic treatment, and most will have had previous experience with children of parents with psychiatric disorders. The clinicians performing the intervention will receive continuous supervision from a family therapist, who is certified in the Family Talk Intervention. In cases of possible mental health problems in the children, the clinicians will consult a child and adolescent psychiatrist who is affiliated with the project. The service as usual arm of the study contains no intervention from the study group.

#### Interventions: concomitant care {11d}

The participants in the study are permitted to participate in other interventions during the time of the interventions such as services within Child Protection Services and peer groups for children of parents with mental illness. Most patients will receive concomitant individual psychotherapeutic treatment during the time of interventions.

#### Provisions for post-trial care {30}

No provisions for post-trial care are planned. Patients that are already in treatment in secondary health services can continue treatment when participating in the trial and after its completion. At baseline, 4 months’ follow-up, 12 months’ follow-up or in the Family Talk Intervention, the families are referred to CAMHS, AMHS or child protection services if needed.

### Outcomes {12}

The complete test battery concerning the child, parents and family is listed in Table 1. The assessments are supervised by a peer group including a clinical professor in child and adolescent psychiatry and psychologists.

**Table 1:**
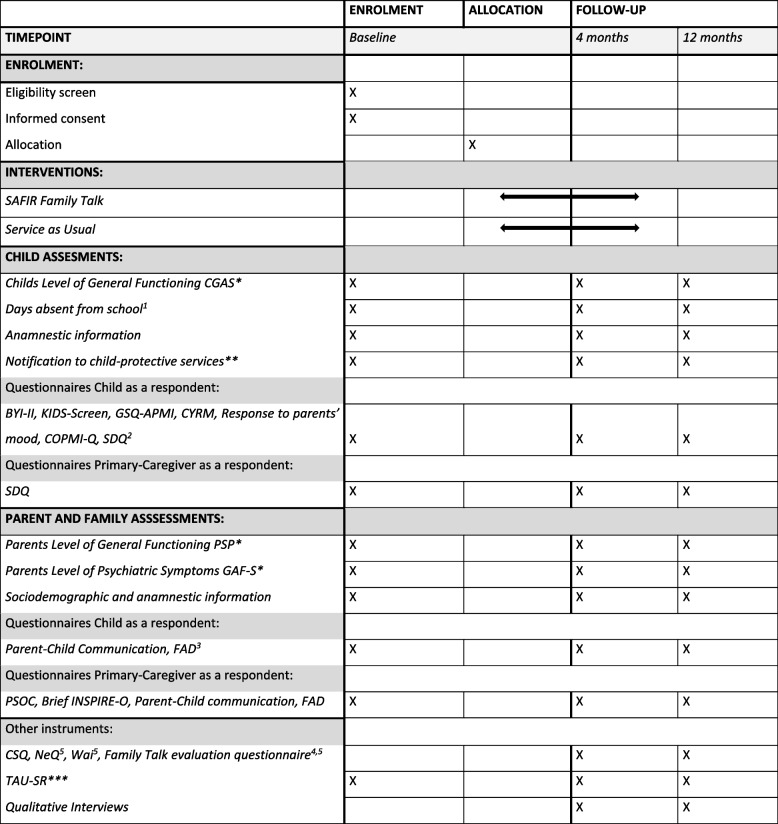
SPIRIT figure

#### Primary outcomes

Change in the Children’s Global Assessment Scale (CGAS) [25] from baseline to 4 and 12 months after baseline is the primary outcome measure concerning the child’s level of functioning. The CGAS is a scale from 1 to 100 (higher scores indicate better functioning), which is included in the diagnostic interview Kiddie Schedule for Affective Disorders and Schizophrenia Present and Lifetime (K-SADS-PL) [26]. It concerns the child’s daily level of functioning in the family, in school and during leisure time. The CGAS has been shown to have high validity and acceptable interrater reliability. It is a dimensional and detailed measurement that accommodates the finding that a given diagnosis can have a very different impact on the functioning of different children. Thus, the CGAS is an ideal tool for capturing any changes the child may experience. CGAS will be measured at baseline, at the end of the intervention and 12 months after baseline, where both the primary caregiver and the child are interviewed. The CGAS score will be rated by blinded assessors with experience in child and adolescent mental health services, and with training and experience in the use of CGAS.

Change in the Family Assessment Device (FAD) [27] (Parent-rated) from baseline to 4 and 12 months after baseline is the primary outcome measure concerning family functioning. The FAD is a thorough questionnaire with 60 items based on a comprehensive sociological theory about the different functions of a family: family problem-solving, communication, roles, affective responsiveness and involvement, behaviour control and general functioning. The FAD allows for a comprehensive picture of family functioning in multiple areas, and through repeated assessments, it can provide insight into whether family members experience improvement in the well-functioning of their family unit. Each item is scored on a 4-point scale according to the extent of which it describes the family. The FAD is completed by the parents at baseline, 4 months and 12 months after baseline and measures each individual’s perception of his or her family.

Change in Parental Sense of Competence (PSOC) from baseline to 4 and 12 months after baseline is the primary outcome measure concerning parents’ overall experiences of competence in their parenting role [28]. The PSOC is a 16-item self-reporting questionnaire measuring parental competence on two dimensions: parental satisfaction and parental self-efficacy. The efficacy factor examines the parents’ competence, capability levels and problem-solving abilities in their parental role, whereas the satisfaction factor examines the parents’ anxiety, motivation and frustration. The PSOC scale was selected as it is a frequently used tool in assessing parenting self-evaluations and has substantial strengths including good content validity. Through repeated assessments, the PSOC can provide insight into whether the parents in the SAFIR Family Talk group feel supported in their parenting skills. Each parent completes the PSOC thinking only of their youngest child of age 7–17 (i.e. the child selected for assessments). Each item is scored on a 6-point Likert scale ranging from 1 (strongly agree) to 6 (strongly disagree). The PSOC is completed by the parents at baseline, 4 months and 12 months after baseline.

#### Secondary outcomes

Secondary outcome measures include change in Beck’s Youth Inventories (BYI-II) [29]: A 99-item self-report questionnaire assessing symptoms of depression, anxiety, anger, disruptive behaviour and self-concept, completed by the child at baseline, 4 months and 12 months after baseline. Each item is rated along a 4-point Likert scale (“never”, “sometimes”, “often” or “always”) and higher scores are associated with negative affect. Also included is change in the Parent–Child Communication questionnaire (Child-rated): A 10-item questionnaire for children of ages 8–12 and 19 items for children aged 13–17 assessing communication between the child and the mentally ill parent. Each item is rated on a 6-point Likert scale, in which a higher score indicates better communication. The Parent–Child Communication questionnaire is completed by the child at baseline, 4 months and 12 months after baseline. Another secondary outcome measure is change in the Response to Parents’ Mood questionnaire assessing the child’s reaction to the parents’ mood. Furthermore, a secondary outcome measure is change in the Brief INSPIRE-O [30]: A 5-item self-report questionnaire completed by the parents assessing personal recovery. Each item is rated on a score from 0 to 100, where a higher score indicates better recovery.

#### Explorative outcomes

Exploratory outcome measures include change in the Strengths and Difficulties Questionnaire (SDQ) [31], the Kidsscreen-27 questionnaire [32], the Child and Youth Resilience Measurement (CYRM) [33], the Guilt and Shame Questionnaire (GSQ-AMPI) [34], the Personal and Social Performance Scale (PSP) [35], the Global Assessment of Functioning (GAF-S) [36], the Parent–Child communication questionnaire (Parent-rated), the Family Talk Evaluation Questionnaire, the Working Alliance Inventory (WAI-SR) [37], the Client Satisfaction Questionnaire (CSQ-8) [38] and the Negative Effects Questionnaire (NeQ) [39], number of days the child was absent from school (information will be obtained from registry and parents’ report), the Family Assessment Device (FAD) [27] (children self-report) and the Children of Parents with Mental Illness Questionnaire (COPMI-Q).

Schedule of enrolment, interventions and assessments (SPIRIT figure). *Semi-structured interview, **self-report on whether there has ever been made a notification to child-protective services concerning the child’s well-being, ***self-report on use of treatment and intervention facilities in private and public institutions (by any family member). ^1^Days of absence from school (registry and parents’ report). ^2^SDQ, Strengths and Difficulties Questionnaire, is only self-report in children ages 11–17. ^3^ FAD, Family Assessment Device, is only self-report in children ages 12–17. ^4^ The Family Talk Evaluation Questionnaire is administered to both children and parents. ^5^ Only administered to families from the SAFIR Family Talk Intervention group. *BYI-II* Beck Youth Inventories Second Edition, *COPMI-Q* Children of Parents with Mental Illness Questionnaire, *CSQ* Client Satisfaction Questionnaire, *CYRM* Child and Youth Resilience Measurement, *FAD* Family Assessment Device, *GAF-S* Global Assessment of Functioning Symptoms scale, *GSQ-APMI* Guilt and Shame Questionnaire for Adolescents of Parents with Mental Illness, *NeQ* Negative Effects Questionnaire, *PSOC* Parental Sense of Competence, *PSP* Personal and Social Performance Scale, *SDQ* Strengths and Difficulties Questionnaire, *TAU-SR* Treatment as Usual Self-Report, *WAI* Working Alliance Inventory.

### Participant timeline {13}

**Fig. 2:**
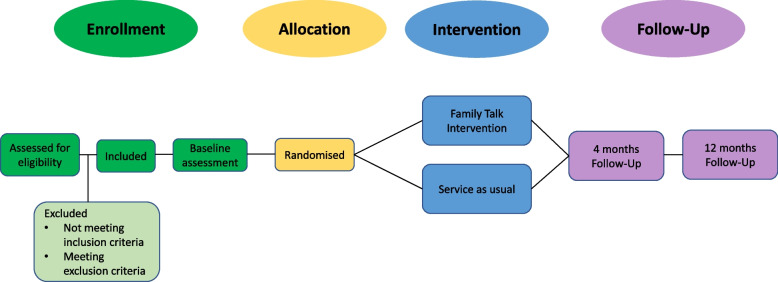
Participant timeline for participants in the SAFIR FAMILY TALK study

Assessment at baseline includes an interview and a battery of questionnaires for both the referred parent with mental illness (called index parent), the other parent and the youngest child in the family between the ages of 7 and 17. After assessment, each family is allocated to either Family Talk or service as usual. Follow-up assessments are conducted at 4 months and 12 months after baseline (see Fig. 2).

Due to COVID-19, the timeline for follow-up assessments was prolonged for some families. Ultimo 2020, primo 2021 and ultimo 2021 relatives of patients were not allowed in the clinic, and therefore assessments were either rescheduled for a later time or done via TEAMS or telephone.

### Sample size {14}

The primary outcome for the children is the change in the estimate of the child’s general functioning. If the intervention results in an increase of the CGAS score of 5 points compared to service as usual (e.g. from 65 to 70, SD = 13), power calculations show that by including 143 children in each group we will be able to measure a difference of 5 points on the CGAS score between the two groups with a power of 0.90. Allocation is 1:1 resulting in *n* = 286.

Power calculations for the primary outcome of parenting sense of competence (PSOC) are based on the mean of the total scale for mothers in a normative population, which is 60.92 with a standard deviation of 8.94 [40]. For a mean difference of 5 points, a power of 0.90 and an error 1 rate of 0.05, a total of 134 participants must be enrolled.

The primary outcome concerning family functioning is the Family Assessment Device (FAD). The mean of general functioning in a normal population is 1.79, with a standard deviation of 0.42 [41]. The cut-off value for familial dissatisfaction is 2.00 [27]. A power calculation with a power of 0.90 and an error 1 rate of 0.05 results in a total of 168 participants.

### Recruitment {15}

Families are primarily recruited from all Mental Health Centers in the Capital Region of Denmark, though all eligible families, whether they are self-referred or via the municipality, are invited to participate. Patients meeting the inclusion criteria are asked by clinicians if a researcher from SAFIR could contact them. If the family consists of people other than the legal parents, they are invited to participate in the assessment and intervention as well. From December 2021, individuals matching the criteria for the parent with a mental illness are identified through the Danish, population-based registers and receive an invitation by electronic mail. An estimated 5% of invited individuals reply to invitations.

Due to COVID-19, the recruitment of patients was very difficult in 2020 and part of 2021. Assessments were cancelled due to illness, and it was very difficult for the research team to create awareness about the project, since oral presentations of research projects were limited or suspended at different out-patient clinics in the Capital Region of Denmark.

## Assignment of interventions: allocation

### Sequence generation {16a}

Families who provide written informed consent are randomly allocated to either Family Talk or service as usual. Randomization will be stratified by site using REDCap software. REDCap is an electronic data capture tool hosted at CIMT in the Capital Region of Denmark. The randomization programme is set up by CH. When the baseline assessment is completed, their contact information is sent to the allocation team that will assign the participants to either intervention or service as usual. The allocation is randomized and computer-generated. The randomization cannot be influenced by the person making it or any other person.

### Concealment mechanism {16b}

Personnel who are not blind to the treatment arm are responsible for the randomization process. When a family has been recruited for the study and baseline assessment has been conducted, the assessor informs the person responsible for conducting the randomization process via e-mail.

Randomization is centralized and computerized with a concealed randomization. Block size will be unknown to the researchers and clinicians. The randomized intervention allocation is concealed until the statistical analyses of the resulting data have been completed and conclusions have been drawn.

### Implementation {16c}

Randomization is carried out by a member of the research team at the Copenhagen Research Center for Mental Health (CORE) who generates the allocation sequence and assigns participants to interventions.

## Assignment of interventions: blinding

### Who will be blinded {17a}

Outcome assessors, data analysts and researchers will be blinded throughout the study, including during the statistical analysis. Due to the nature of the intervention, participants and therapists performing the Family Talk Intervention are not blinded.

### Procedure for unblinding if needed {17b}

The families are instructed in advance not to reveal allocation to researchers at 4- and 12-month follow-up assessments. If unblinding occurs, it will be registered and another assessor, blind to treatment allocation, will perform the outcome assessment at the follow-up. If this is not possible, the final outcome scores will be set by a blinded rater or assessor.

## Data collection and management

### Plans for assessment and collection of outcomes {18a}

The families are assessed with a range of interviews and questionnaires at baseline, at 4-month follow-up and at 12-month follow-up (see test battery, Table 1). The baseline assessment takes approximately 3 h to complete, and each follow-up assessment takes approximately 2.5 h. To ensure the quality of the data, the assessors are trained in administering both the Personal and Social Performance scale (PSP) (Morosini et al., 2000), the General assessment of function—Symptoms (GAF-S) [36], the Children’s Global Assessment Scale (CGAS) [25] and each of the different questionnaires. Both the PSP, the GAF-S and the CGAS ratings are made as consensus.

### Plans to promote participant retention and complete follow-up {18b}

The participating families will be contacted by telephone before planned follow-up interviews. If preferred by the families, the next follow-up is often planned in combination with the current. Assessors are flexible and can rearrange the scheduled time if needed. Patients who are in an unstable condition can be assessed at home and both the 4- or 12-month follow-up with the parent can be conducted by telephone to make it more manageable for the families. A taxi can be arranged when needed.

All families receive a gift certificate, adding up to 1000 DKK if all 3 assessments are completed.

Combined with a pragmatic approach, and an emphasis on the importance of participating in research, this hopefully keeps enrolled families in the study and makes them complete follow-up interviews.

During COVID-19, assessments were either done by telephone or TEAMS or rescheduled for a later time.

### Data management {19}

All data including personal information about enrolled participants is collected by the assessors during the interview in the secure web application for building and managing online surveys and databases, REDCap [24]. The surveys for the parents are either answered on-site or at home via links that send data directly to the database. The surveys for the children are either read out or filled in independently on-site depending on the child’s age, ability to read and comprehension of the questions. Data obtained in Family Talk Intervention arm by the trained professionals will also be collected in REDCap. REDCap has a complete audit trail on all data transactions, detailed user rights and access control management complying with Danish legislation (Databeskyttelsesforordningen). Only assigned researchers can access REDCap which contains all data. All written statements of consent are kept in a locked file cabinet.

### Confidentiality {27}

Data collected during the research trial will be kept strictly confidential and only accessed by members of the trial team. All participants will be allocated an individual trial identification number. Research data will be exported from REDCap without personal identifiers. Only AR, LJM and SN will have full access rights to the full data set while the trial is running. On completion, CH will be given access to make the statistical analysis. No plans are made in regard to sharing anonymous data with other researchers outside the trial unit.

### Plans for collection, laboratory evaluation and storage of biological specimens for genetic or molecular analysis in this trial/future use {33}

See above 26b; there will be no biological specimens collected.

## Statistical methods

### Statistical methods for primary and secondary outcomes {20a}

Tests will be two-tailed. The primary outcome analysis will be an intention-to-treat (ITT) analysis. Analysis of covariance (ANCOVA) will be used to calculate any significant results between the two groups, using the baseline value and the gender of the child as stratification variables.

### Interim analyses {21b}

There will be no interim analyses.

### Methods for additional analyses (e.g. subgroup analyses) {20b}

Subgroup analyses will explore whether parent’s PSP and GAF-S influence both primary and other secondary outcomes. The same will be explored for children’s C-GAS. Subgroup analyses will explore whether parent’s PSP and GAF-S or children’s C-GAS influence drop-out rates or show-up rates in the intervention group. The cut-off scores for subgroups have not been decided.

### Methods in analysis to handle protocol non-adherence and any statistical methods to handle missing data {20c}

Missing data are analysed according to the intention-to treat principles, i.e. analysing individuals to their allocated groups regardless of, e.g. protocol non-adherence. Multiple imputations will be used to handle missing data. The imputations will be based on a linear regression model with 100 imputations and 20 iterations. The pooled analyses will subsequently be used for our analysis. As predictors in the imputation model, we will select variables if they are independent predictors of the outcome or predictors of missing data (*P* < 0.05 in a univariate model).

### Plans to give access to the full protocol, participant-level data and statistical code {31c}

The document constitutes the full trial protocol. Following completion of the trial, datasets and statistical code used in this study will be available from the corresponding author on reasonable requests.

## Oversight and monitoring

### Composition of the coordinating centre and trial steering committee {5d}

Responsibilities of Trial Steering Committee: AR, NR, CH, MN and AT review the progress of study and necessary changes to the protocol to facilitate the smooth running of the study.

Responsibilities of Trial Management Committee: AR, LJM, SN, CH and NR are responsible for the recruitment and management of participants in the clinical setting, budget administration and contractual issues with individual centres.

### Composition of the data monitoring committee, its role and reporting structure {21a}

This study does not have a data monitoring committee. All participants randomized to the Family Talk intervention will be monitored for any harmful effect by the clinician who provides the intervention. If necessary, the principal investigator will make the final decision to terminate the trial.

### Adverse event reporting and harms {22}

Parents in the Family Talk Intervention group answer the Negative Effects Questionnaire (NeQ) [39] investigating the negative effects of the intervention.

### Frequency and plans for auditing trial conduct {23}

The Trial Steering Committee are responsible for the auditing of the trial and meet every third month throughout the study period.

During the COVID-19 lockdown, the Trial Steering Committee had online meetings every week in order to adapt the planning and execution of the trial.

The auditing is independent from the sponsors of the trial.

### Plans for communicating important protocol amendments to relevant parties (e.g. trial participants, ethical committees) {25}

All changes will be communicated to sponsors and other relevant parties. In addition, deviations from the published protocol will be documented in the trial registration on ClinicalTrials.gov (Unique Protocol ID: 127,849).

### Dissemination plans {31a}

The researchers will communicate trial results to participants, healthcare professionals, the public and other relevant groups via publication in peer-reviewed journals, scientific meetings and public talks.

## Discussion

In this paper, we have described the planned investigation of the effect of Family Talk Preventive Intervention versus service as usual for families where parents have a mental illness—The SAFIR project—which is to be carried out in the context of the Danish Mental Health Services. Prevention in mental health is a relatively under-developed area especially in comparison with prevention in physical health [17]. Given the magnitude of the public health problem of mental illness, as well as the tremendous economic costs to society and the associated personal suffering, it is striking that mental illness is still almost exclusively treated as it arises. A basic principle of prevention is that early intervention is more efficient and requires fewer resources than treating an established illness. This principle is the foundation of Family Talk as developed by Beardslee over 40 years ago. As of 2021, the SAFIR initiative is in line with the strategic approach proposed by Arango et al. [17] in which different populations are triaged for preventive intervention according to their level of risk for mental illness. According to this hierarchy, children of parents with mental illness require selective primary preventive intervention which should be “effective and associated with low risk of adverse events and moderate costs”. Indeed, Family Talk is associated with moderate costs and has been documented to have a low prevalence of adverse events [42], but the effects of the intervention are still unclear. One challenge to prevention research is the choice of appropriate measurements to document effects, and in mental health research, the development of such measurements is still immature. The expected effects are on a continuum of the endpoint, i.e. future mental illness in the children, to more intermediate effects during and shortly after the intervention. To document short-term effects on the endpoint is unrealistic as most children will not have developed a mental illness regardless of a preventive intervention. While instruments for measurement of the presence and severity of mental illness are well developed, determining the immediate and short-term effects faces different challenges: Which factors can be expected to change on short term due to the preventive interventions? For whom will the change take place (children and/or parents) and which instruments are best suited to document this change? Those are crucial questions. For the most part, previous RCTs on Family Talk have not been successful in documenting effects on measures of clinical or subclinical symptoms of mental health problems and behaviour [7], suggesting that such measures are not sensitive to short-term effects of the intervention. Also, in order to measure effects on psychiatric diagnosis of the children, a very large sample size is needed. In Germany [20], a similar Family talk intervention study plans to investigate children’s mental health determined with Kiddie-SADS-PL as the primary outcome, requiring an estimated sample size of 800 families to show a significant change in psychopathology in the child over an 18-month follow-up period. The most prominent effects in previous trials have been noted for changes in behaviour such as talking about mental illness (children and parents) and attitudes concerning mental illness such as reduction in feelings of shame and guilt (parents). However, the longest follow-up time was 4.5 years [42], and thus it has not been investigated if these factors can be used as reliable indicators of risk reduction in terms of mental illness in the children. Due to the scientific challenge in preventive psychiatry, we chose intermediate measurements concerning the child, the parent(s) and the family. Level of functioning, C-GAS, can detect change both within the normal, subclinical and clinical areas of functioning, thus avoiding floor and ceiling effects in the data. This is important, as only a few children will have developed any psychiatric symptoms or be in acute need of Child Protection Services during the 1-year follow-up. It is more likely that the children’s level of daily functioning will change over this follow-up interval. The Parental Sense of Competency questionnaire, which is a validated instrument, was chosen to measure parents’ experiences of competency in their parenting role, the rationale being that, according to the theory of change in Family Talk, positive changes in children take place through changes in their parents.

### Strengths

Our study will provide an important contribution to the international evidence base for the Family Talk Intervention in numerous ways, including the RCT design, the number of families included from a help seeking, clinical population, the modifications of the method to include families with any mental illness and our primary outcomes, shedding light on new aspects of the effects of the intervention.

The national Danish registers make long-term follow-up of the SAFIR cohort possible in the future to examine whether the intervention had an impact on the development of mental disorders and other important indicators of adult adjustment in the children. The unique personal identification number assigned to all live-born children and new residents in Denmark since 1968 can be used to link data from the SAFIR cohort to different registers with information on psychiatric diagnoses, socioeconomic position, etc., on an individual level [43]. It will also be possible to contact parents and children again and gain new information through surveys and interviews.

One of the key strengths is the sample size of 286 participating families—an ambitious but realistic goal. Outcomes from the four earlier randomized controlled trials have included between 28 and 109 participating families [8, 44].

In our study, the method is extended to include patients with any psychiatric disorder, building upon the findings of the Swedish study which included 103 parents with either depression, psychosis, personality disorder or eating disorder [9]. Their findings support the assumption that Family talk is safe and feasible in transdiagnostic psychiatric populations but was based on a rather small sample size. A further strength of our study is the many measures where the child is the informant. Whereas for instance Solantaus and colleagues used the Strengths and Difficulties Questionnaire as informed by the parents [8], we chose measures where the child is the informant to avoid possible bias from the parents associated with factors such as parental psychopathology and to obtain information directly from the child. The inclusion of the other parent, i.e. the parent who is not a patient, is also important as previous studies [45] suggest that they often feel unsupported by mental health services regarding the care that they provide and would like services to consult and involve them more in the treatment process, considering the other parent is also important as higher prevalence of mental illness and lower levels of functioning have been noted for individuals who had a child by a partner with a mental illness [46].

### Limitations

Many difficulties in recruitment have been described in a study on preventive programmes for children of parents with mental illness [47]. Clinicians’ workload is an important issue as they drive the referral of families to this study. Parental double stigma (i.e. mental illness and struggling with parenting) might prevent families from participating, perhaps due to a fear of being judged as unfit parents or of having the children removed by social services.

A common problem in all clinical trials is how to avoid attrition, in our case especially in the control group of families who did not receive Family Talk. As a means to keep families in the trial, gift certificates are offered at follow-up assessments. It is also made clear that the contributions made by the family in participating in follow-up are of great importance in gaining knowledge on how to prevent future mental illness.

Another possible limitation is that of a selection bias in the parents who chose to participate in the trial and a non-representative population of parents with mental illness, for instance, a selection where parents with either relatively high or low levels of psychiatric symptoms or functioning might choose to participate. In that case, the results of the study might not be applicable to a general population of parents with mental illness.

## Trial status

Recruitment began on September 15, 2020, and is expected to be completed in December 2024. As of January 13, 2023, 180 families have been randomized to either Family Talk or service as usual.

## Data Availability

AR, LJM, SN and CH will have access to the final data set. Any data required to support the protocol can be supplied upon reasonable request from the corresponding author.

## Abbreviations

SAU: Service as usual
CAMHS: Child and adolescent mental health services
AMHS: Adult mental health services
